# Dealing with missing outcome data in meta‐analysis

**DOI:** 10.1002/jrsm.1349

**Published:** 2019-06-09

**Authors:** Dimitris Mavridis, Ian R. White

**Affiliations:** ^1^ Department of Primary Education, School of Education University of Ioannina Ioannina Greece; ^2^ Sorbonne Paris Cité, Faculté de Médecine Paris Descartes University Paris France; ^3^ Institute of Clinical Trials and Methodology, MRC Clinical Trials Unit University College London London

**Keywords:** informative missingness odds ratio, informative missingness parameter, meta‐analysis, missing data, missing not at random

## Abstract

Missing data result in less precise and possibly biased effect estimates in single studies. Bias arising from studies with incomplete outcome data is naturally propagated in a meta‐analysis. Conventional analysis using only individuals with available data is adequate when the meta‐analyst can be confident that the data are missing at random (MAR) in every study—that is, that the probability of missing data does not depend on unobserved variables, conditional on observed variables. Usually, such confidence is unjustified as participants may drop out due to lack of improvement or adverse effects. The MAR assumption cannot be tested, and a sensitivity analysis to assess how robust results are to reasonable deviations from the MAR assumption is important. Two methods may be used based on plausible alternative assumptions about the missing data. Firstly, the distribution of reasons for missing data may be used to impute the missing values. Secondly, the analyst may specify the magnitude and uncertainty of possible departures from the missing at random assumption, and these may be used to correct bias and reweight the studies. This is achieved by employing a pattern mixture model and describing how the outcome in the missing participants is related to the outcome in the completers. Ideally, this relationship is informed using expert opinion. The methods are illustrated in two examples with binary and continuous outcomes. We provide recommendations on what trial investigators and systematic reviewers should do to minimize the problem of missing outcome data in meta‐analysis.

## 1 INTRODUCTION

Missing outcome data are a common occurrence even in well‐conducted randomized clinical trials (RCTs). They may compromise the validity of the analysis of a single study^1^ and are consequently a threat to the validity of a meta‐analysis. The threat has been neglected in the meta‐analysis literature as researchers typically assume that the problem has been dealt with at the trial level. This manuscript explores what we may know about missing data, describes the analysis options in single studies, discusses the methods available in meta‐analysis, and makes suggestions for practice, with a primary focus on aggregate data (AD) meta‐analysis.

The term “missing data” has various meanings in systematic reviews. In this manuscript, we use the term to refer only to missing outcome data and not to missing studies, missing statistics, or whole outcomes not reported in a study. We consider that in some (or all) of the studies, some participants did not provide any outcome data. We discuss the issues in the context of RCTs and without adjustment for baseline covariates.

## 2 ANALYSIS OF A SINGLE STUDY WITH MISSING DATA

Αn RCT is the gold standard for testing the efficacy of an intervention. Randomization ensures that prognostic factors are distributed equally across arms and any systematic difference in the outcome can be attributed to the intervention received. Missing data arise, for example, if participants drop out. Under certain circumstances, missing data may introduce bias and yield misleading conclusions. The problem is well recognized, and many methods have been suggested to account for missing data in RCTs.^2^, ^3^, ^4^, ^5^

The intention to treat (ITT) principle requires all participants in an RCT to be included in the analysis in the arm to which they were randomized. An ITT analysis preserves randomization and avoids bias introduced by dropout and noncompliance.^6^ However, there is no consensus in the literature on how to perform ITT analysis when outcomes are missing.^7^ Some authors argue that the ITT principle requires missing values to be imputed, using methods such as last observation carried forward (LOCF) or multiple imputation.^8^

From a statistical perspective, any analysis of a study with missing data makes an assumption about the missing data. A principled approach starts by considering what assumption is plausible and hence chooses a suitable primary analysis.^9^ The validity of the analysis rests on the plausibility of its assumptions, not on whether or not missing values were imputed. Sensitivity analyses are then needed to explore how robust the results are to plausible deviations from the assumption in the primary analysis. These ideas lead to an ITT analysis strategy, which emphasizes the inclusion of all randomized participants in sensitivity analyses.^10^

Assumptions about missing data are often described using Rubin's framework,^11^ which describes the various missing data mechanisms and the relationships between variables (observed and unobserved) and the probability of missing data. Data are missing completely at random (MCAR) if the probability of missing data does not depend on observed or unobserved variables. In this case, missing data have the same distribution as observed data. For example, blood pressure data are likely to be MCAR if they are missing because of breakdown of an automatic sphygmomanometer.^4^ Data are missing at random (MAR) if missing data have the same distribution as observed data, conditional on other variables included in the analysis. For example, blood pressure data are likely to be MAR if age, but no other factor, predicts blood pressure measurement. Typically, older people would have higher blood pressure levels, but conditioning on age, MAR holds if people with high and low blood pressure are equally likely to have their blood pressure measured. Finally, if data are not MAR then they are missing not at random (MNAR) or informatively missing (IM). MNAR means that the probability of missing data depends on unobserved variables, usually the outcome itself. For example, blood pressure data are MNAR if, within age groups, the outcomes for participants who dropped out are worse than the observed outcomes. Other assumptions that do not fit neatly into the MCAR/MAR/MNAR framework are possible: for example, the assumption underlying a LOCF analysis is that missing values do not differ on average from last observed values.

In practice, the starting point of an analysis is usually to ignore missing data in an available case analysis (ACA), also called a complete case analysis. This assumes that data are MAR. If instead the data are MNAR, then ACA risks bias in the intervention effect, especially if dropout rates vary between arms.^12^

Several approaches have been suggested to handle missing data in clinical trials. Some of the most popular methods are summarized in Table 1.

**Table 1:** Methods for handling missing outcome data in clinical trials

Method		Description	Assumptions About Missing Outcome Data	Use in Meta‐analysis
Available case analysis		Ignores missing participants	MAR	Common starting point in AD and IPD meta‐analysis
Single imputation methods for binary data				
	Impute failure	Imputes missing values as failures	Always failures	Possible starting point in AD and IPD meta‐analysis (eg, smoking cessation trials)
	Worst (best)‐case scenario	Imputes failures in the treatment arm and successes in the control (or vice versa)	Always failures or always successes, depending on arm	Extreme assumption in AD and IPD meta‐analysis that may be useful in sensitivity analysis
Single imputation methods for all data				
	Last observation carried forward	Imputes missing values with the participants' last observation	The missing value for a participant has the same mean as the last observed value	Often used in trial reports and hence also in AD meta‐analysis; can be avoided in IPD meta‐analysis. Usually an unrealistic assumption; can underestimate uncertainty^13^
	Single imputation	Imputes missing values, usually borrowing information from observed outcomes (not necessarily from the same arm or study)	Missing values equal a prespecified value without uncertainty	Does not take uncertainty in the imputed values into account
Methods that take uncertainty into account				
	Multiple imputation	Builds a model to predict missing outcome from the participants' observed outcome, and adds appropriate random error^14^	MAR	Useful in IPD meta‐analysis but rarely used with AD
	Likelihood methods	Fits a model to the observed data	MAR	Useful in IPD meta‐analysis but rarely used with AD
		Fits a model to the observed data and the probability of being missing	MNAR	Hard to implement but potentially useful in IPD meta‐analysis
	Pattern mixture model	Builds a model for the outcome conditional on whether it is missing or not and a model for the missingness mechanism^12^	Addresses departures from the MAR assumption (MNAR)	Useful in AD and IPD meta‐analysis. The relation between missing and observed outcomes can be informed by expert opinion or by a sensitivity analysis

## 3 META‐ANALYSIS WITH MISSING DATA

Inappropriate analysis with missing data in RCTs leads to biased meta‐analytic estimates. The meta‐analyst therefore faces four tasks, which we discuss in turn.

### 3.1 Understand the extent of missing data in each included study

Standard data extraction yields the number of individuals analyzed in each arm, with summary statistics (count for binary outcomes, or mean and standard deviation for continuous outcomes). To allow for missing data, we also need to know at least the number of study participants with missing data in each arm. The CONSORT statement expects reporting of the number of participants who were randomly assigned and the number of participants in each arm included in each analysis.^15^ Surveys have shown that 95% of trials in major medical journals report some missing outcome data^16^ and 94% of palliative care trials report the number of participants not included in the primary outcome analysis.^17^ Systematic reviews have lower rates of reporting numbers of participants with missing data—47% of Cochrane reviews and 7% of non‐Cochrane reviews.^18^

If possible, the number of missing values in each arm should be broken down by the reasons for the data being missing: for example, how many were due to loss to follow‐up (which may be plausibly MAR) and how many were due to disillusioned patients withdrawing from a trial (which are likely to be MNAR, with worse outcomes than those observed). The meta‐analyst needs to define a classification of reasons to make results comparable between studies. When the outcome of the review is a trial's secondary outcome, it may be necessary to use reasons reported for the trial's primary outcome, which are likely to be better reported.

### 3.2 Understand how the missing data were handled in each published report

The quality of published analyses can be hard to judge: Studies typically report results from ACA or from some simple imputation method, but reporting of methods used can be poor. For example, in 2000, only 34% of studies in PubMed reported the handling of missing data,^19^ but by 2013, methods could be classified in 100% of trials in major medical journals.^16^

Errors can arise through misunderstanding how data were handled. For example, a meta‐analysis of effectiveness of brief interventions targeting excessive drinkers in general practice set out to regard missing values as failures (thus giving a lower bound to the success rate)^20^ but was overzealous: One study's reported results included all participants, with missing values imputed as failures, but the reviewers took this study as reporting only available cases and applied a further correction.^21^

### 3.3 Evaluate the risk of bias due to missing data in each published report

Risk of bias due to missing data is included in the Cochrane risk of bias tool.^22^ The original version of the Cochrane tool asks assessors to describe the completeness of outcome data for each outcome, the numbers in each intervention arm (compared with total randomized participants) and the reasons for attrition or exclusions. Participants in a focus group felt that assessing the risk of bias due to incomplete outcome data was more difficult to assess than other biases.^23^

### 3.4 Perform alternative analyses exploring the impact of the missing data under different assumptions

Valid statistical methods are needed to account for missing outcome data in the meta‐analysis, and several methods have been suggested.^24^ As well as correcting for bias in individual studies and inflating the standard error of the pooled estimate to allow for uncertainty about missing data, we also aim to change the weights assigned to studies to reflect which studies are more uncertain. Studies with high missing rates should be penalized relatively more when pooled in a meta‐analysis because their effect estimates may be biased (under MNAR).

The primary analysis is commonly an ACA: A sensitivity analysis is then needed to explore the impact of departures from the MAR assumption implied in an ACA on the point estimate and its standard error. The methods we propose are primarily intended to be used in such a sensitivity analysis. However, in a meta‐analysis where bias from missing data was a serious concern, the methods proposed could form a primary analysis.

We assume we have access only to AD, so we cannot use all the methods presented in Table 1 (eg, multiple imputation). If we have individual participant data (IPD), suitable methods from Table 1 can be used to analyze each study, as we note below; the methods described here would be less appropriate for primary analysis but would be useful in sensitivity analyses.

Spineli et al investigated 140 systematic reviews in mental health published in the Cochrane library since 2009 and found that only 27 (19%) reported a sensitivity analysis.^25^ They found that 14 of those 27 reviews (52%) considered a best/worst case scenario (13 studies did that only for the experimental arm). They also found that 109 (78%) reviews had at least one study where missing data were imputed using LOCF.

The best/worst case scenarios are typically used as sensitivity analyses but may produce unrealistic results in practice, especially if missing rates are high. Gamble and Hollis suggested that the discrepancy between best‐ and worst‐case scenarios should be used to inform the downweighting of studies with more missing data.^26^ However, because best‐ and worst‐case scenarios are implausible in most meta‐analyses, their method was unrealistically conservative. Methods based on single imputation have also been suggested for meta‐analysis of continuous outcomes,^27^ eg, impute the worst observed mean.

We next describe two improvements on the above methods. In Section 4, we use data on reasons for missing data to improve our analysis. In Section 5, we specify the magnitude of plausible departures from the MAR assumption.

## 4 METHODS 1: USING REASONS FOR MISSING DATA AND SIMPLE ASSUMPTIONS

Our first approach requires data on the distribution of reasons for missing data in at least some studies. The methods described here were proposed for meta‐analyses with binary outcomes.^28^

If reasons for missing data are unreported in some studies then they can be imputed by the within‐arm average across other studies.

The key idea is to consider the individuals in each reason group within each arm and to impute the missing data by making specific assumptions about the missing data mechanism (an imputed case analysis [ICA]). These specific assumptions could involve imputing failures (ICA‐0), imputing successes (ICA‐1), imputing the control arm proportion (ICA‐p_C_), and imputing the arm‐specific proportion (ICA‐p).

ICA‐0 was used for reasons such as lack of therapeutic benefit and ICA‐1 for positive response. ICA‐p_C_ was used for adverse events, because patients with adverse events would withdraw from treatment and therefore might be expected to perform like untreated patients; this implicitly assumed that patients withdrawing from treatment did not differ in any other way from those remaining on treatment. Finally, ICA‐p was used for reasons such as loss to follow‐up, which could plausibly be considered to be MAR. Once imputations have been done, care is needed to obtain correct standard errors: It would be wrong to treat the imputed data as real data, since this would deflate standard errors and give too much weight to studies with missing data as well as overestimating the certainty of the results.^24^

This approach is broad and equally applicable to AD or to IPD subject only to what is known about reasons for missing data. For example, it includes best‐ and worst‐case analyses (by setting ICA‐1 in the treatment arm and ICA‐0 in controls, and vice versa). A further extension is given in Section 5.

## 5 METHODS 2: QUANTIFYING DEPARTURES FROM MAR

The method in Section 4 only allows a limited range of assumptions within each reason group. Now we expand the range of assumptions by quantifying departures from MAR. We do not require data on reasons for missing data, although these can be used as noted later. When data are MNAR, we need to specify a joint model for the observed and missing outcomes and the missing data pattern. There are two popular models for doing so, selection and pattern mixture models.^29^ Pattern‐mixture models use the marginal distribution of the missing data pattern and the conditional distribution of the observed and missing data given the missing data pattern, while selection models use the exact opposite. In this setting, we apply a pattern mixture model where the distribution of the missing outcomes given the observed outcomes and the missing data pattern is specified using prior beliefs about the missing data. Prior beliefs are expressed using an informative missingness parameter (IMP), which relates the mean outcome in the missing data to that in the observed data, for each arm of each trial, and hence expresses the degree of departure from the MAR assumption. The IMP is unknown and cannot be informed by the data: Ideally, expert (clinical) opinion is used to elicit information about likely values of the IMP. These prior beliefs are then incorporated into the analysis in a two‐stage approach.^30^ At the first stage, we compute study‐specific effect estimates and their standard errors adjusted for the prior beliefs about the missing data. At the second stage, the adjusted estimates are combined in a standard meta‐analysis.

With binary outcome data, a suitable IMP is the ratio of the odds of the outcome among participants with missing outcomes to the odds of the outcome among observed participants and is referred to as the informative missingness odds ratio (IMOR).^28^, ^30^ The IMOR approach incorporates the best/worst‐case scenarios as special cases but allows less extreme assumptions. An IMOR of 2 in a beneficial outcome states that the odds of success in the missing participants are double the odds in the observed participants: eg, participants left the study because of early response. An IMOR of 0.5 states that the odds in the missing participants are half the odds in the observed participants: eg, the participants left the study because of lack of improvement. Suppose that we have 100 participants randomized in an arm, of whom 40 recovered, 20 did not recover (odds in observed = 40/20), and 40 did not provide any outcome data. Suppose that an expert believes that only 10 of the 40 unobserved participants would have recovered (odds in missing = 10/30). Then the expert's estimate of the IMOR is the ratio of the odds in missing to the odds in observed and equals 1/6.

With continuous outcomes, the IMP compares the mean in missing participants to the mean in the observed participants.^31^ It may be defined as the informative missingness difference of means (IMDoM) or the informative missingness ratio of means (IMRoM). An IMDoM of 1 states that the mean value in the missing participants exceeds the mean value of the observed participants by one unit. An IMRoM of 1.5 states that the mean value in the missing participants is 1.5 times the mean value in the observed participants. The IMDoM or IMRoM can be elicited by giving an expert the mean value in the observed data and asking for the mean value in the missing data.

In practice, experts should express a range of plausible values of the IMP. These may be used in a sensitivity analysis. For example, if the plausible range of the IMP is from −2 to 2, then the meta‐analysis could be performed with the IMP assumed to be −2 in all arms of all studies and then repeated with −1, 0, 1, and 2. Alternatively, the range of plausible values of the IMP may be viewed as a prior belief distribution specified by a mean IMP and a standard deviation: For example, the IMP above could be taken as normally distributed with mean 0 and standard deviation 1 (so that the expert is 95% sure that the true IMP is within the plausible range). In this approach, a nonzero mean IMP tends to shift the point estimates, while uncertainty about the IMP (expressed through its standard deviation) tends to increase the study‐specific standard errors, with two consequences: Studies with less missing data tend to receive greater weight, and the standard error of the pooled estimate tends to increase. An important extension of the method allows the IMP to differ across treatment arms.^12^

The method has been extended for network meta‐analysis models for both dichotomous and continuous outcomes.^31^, ^32^ The methods of Sections 4 and 5 can be combined so that one category of reasons is imputed with a specified IMP. In principle, a distribution of IMPs could be used for each reason group, but this is not currently available in statistical software. IPD would facilitate more complex analyses, perhaps using multiple imputation with MNAR mechanisms (see, eg, Leacy et al^33^). Alternative fully Bayesian approaches have been proposed.^34^, ^35^

## 6 TWO WORKED EXAMPLES

We illustrate the suggested methods using two meta‐analyses, one with a binary and one with a continuous outcome.

### 6.1 Haloperidol meta‐analysis

We use a meta‐analysis of studies comparing haloperidol with placebo in the treatment of schizophrenia.^36^ The outcome is coded as “success” or “failure” on the basis of clinical improvement. Information about missing values was extracted and analyzed by Higgins and colleagues^28^ and is reproduced in Table 2. Two studies (Beasley 1996, Selman 1976) have particularly large numbers of missing values. This is because other studies imputed missing outcomes using LOCF. We consider common‐effect meta‐analyses for the risk ratio.

**Table 2:** Haloperidol meta‐analysis: main results and reasons for missing data

First Author	Year	Main Results Data						Reasons for Missing Data							
		Haloperidol Arm			Placebo Arm			Haloperidol Arm				Placebo Arm			
		Successes	Failures	Missing	Successes	Failures	Missing	ICA‐0	ICA‐1	ICA‐p_C_	ICA‐p	ICA‐0	ICA‐1	ICA‐p_C_	ICA‐p
Arvanitis	1997	25	25	2	18	33	0	17	0	17	0	30	0	5	0
Beasley	1996	29	18	22	20	14	34	19	0	15	5	32	0	13	1
Bechelli	1983	12	17	1	2	28	1	0	0	0	1	0	0	0	1
Borison	1992	3	9	0	0	12	0	0	0	0	0	0	0	0	0
Chouinard	1993	10	11	0	3	19	0	11	0	2	0	10	0	6	0
Durost	1964	11	8	0	1	14	0	0	0	0	0	0	0	0	0
Garry	1962	7	18	1	4	21	1	0	0	1	0	0	0	1	0
Howard	1974	8	9	0	3	10	0	0	0	0	0	0	0	0	0
Marder	1994	19	45	2	14	50	2	25	0	0	13	41	0	0	4
Nishikawa	1982	1	9	0	0	10	0	0	0	0	0	0	0	0	0
Nishikawa	1984	11	23	3	0	13	0					0	0	0	0
Reschke	1974	20	9	0	2	9	0	0	0	0	2	6	0	0	0
Selman	1976	17	1	11	7	4	18	4	0	0	7	8	0	0	10
Serafetinides	1972	4	10	0	0	13	1	0	0	0	0	1	0	0	0
Simpson	1967	2	14	0	0	7	1	0	0	0	0				
Spencer	1992	11	1	0	1	11	0	0	0	0	0	0	0	0	0
Vichaiya	1971	9	20	1	0	29	1	0	0	0	1	0	0	0	1

*Note*. In some cases, reasons refer to a different outcome.

**Figure 1:**
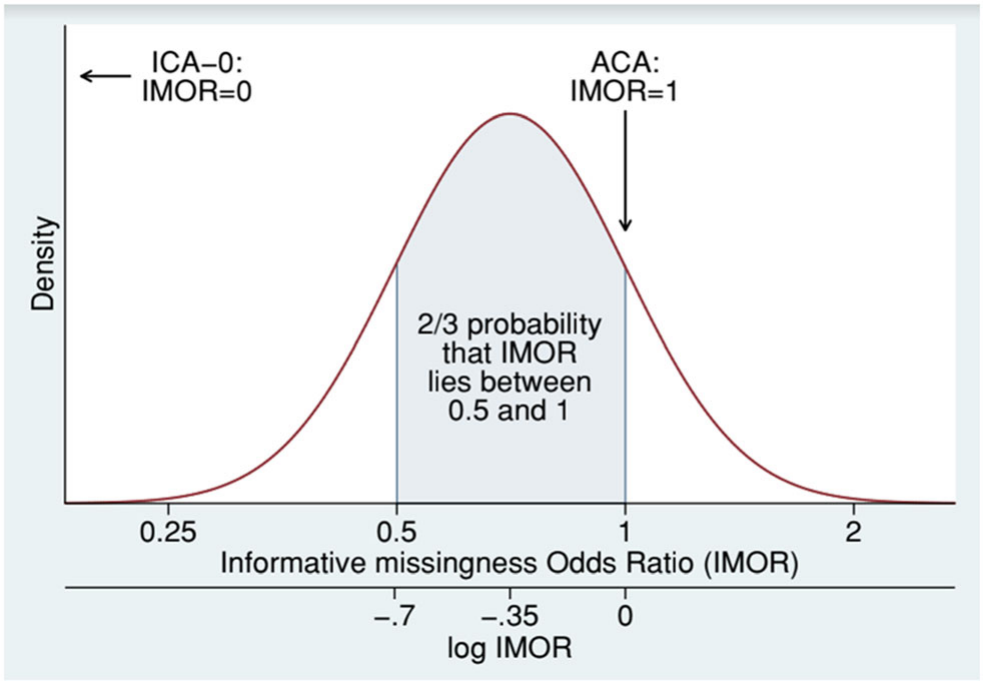
Plausible distribution for the informative missingness odds ratio (IMOR) in the haloperidol meta‐analysis [Colour figure can be viewed at http://wileyonlinelibrary.com]

We present four possible ways of handling the missing data out of a wide possible range. First, an ACA would be the standard choice. However, in this mental health setting, missing values are likely to show less improvement than observed values. A second analysis therefore imputes all missing values as failures (ICA‐0). Because here the outcome is clinical improvement, this may be considered to be an LOCF analysis. However, the truth about the missing data is likely to lie between ACA and ICA‐0. In our third analysis, we express this by using the reasons for missing data given in Table 2. Finally, our fourth analysis expresses uncertainty about the missing data by using a plausible distribution for the IMOR (Figure 1). We imagine that we had asked experts for their views about the missing data, and they had suggested that the odds of success in the missing participants was unlikely to be greater than the odds of the success in the observed participants but also unlikely to be less than half the odds of the success in the observed participants. On further discussion, we imagine that they judged “unlikely” in the above statements to mean a probability of about 1/6. We can translate this to a statement that the IMOR lies below 0.5 with probability 1/6, between 0.5 and 1 with probability 2/3, and above 1 with probability 1/6. Further assuming a normal distribution for the log IMOR, we can derive that the log IMOR has mean (*ln*(0.5)+ *ln* (1))/2  =  − 0.347 and standard deviation (*ln*(1) −  *ln* (0.5))/(2*z*_5/6_) = 0.358, where *z*_5/6_ is the normal deviate with cumulative probability 5/6. This approximates to a normal distribution with mean −0.35 and standard deviation 0.35.

**Figure 2:**
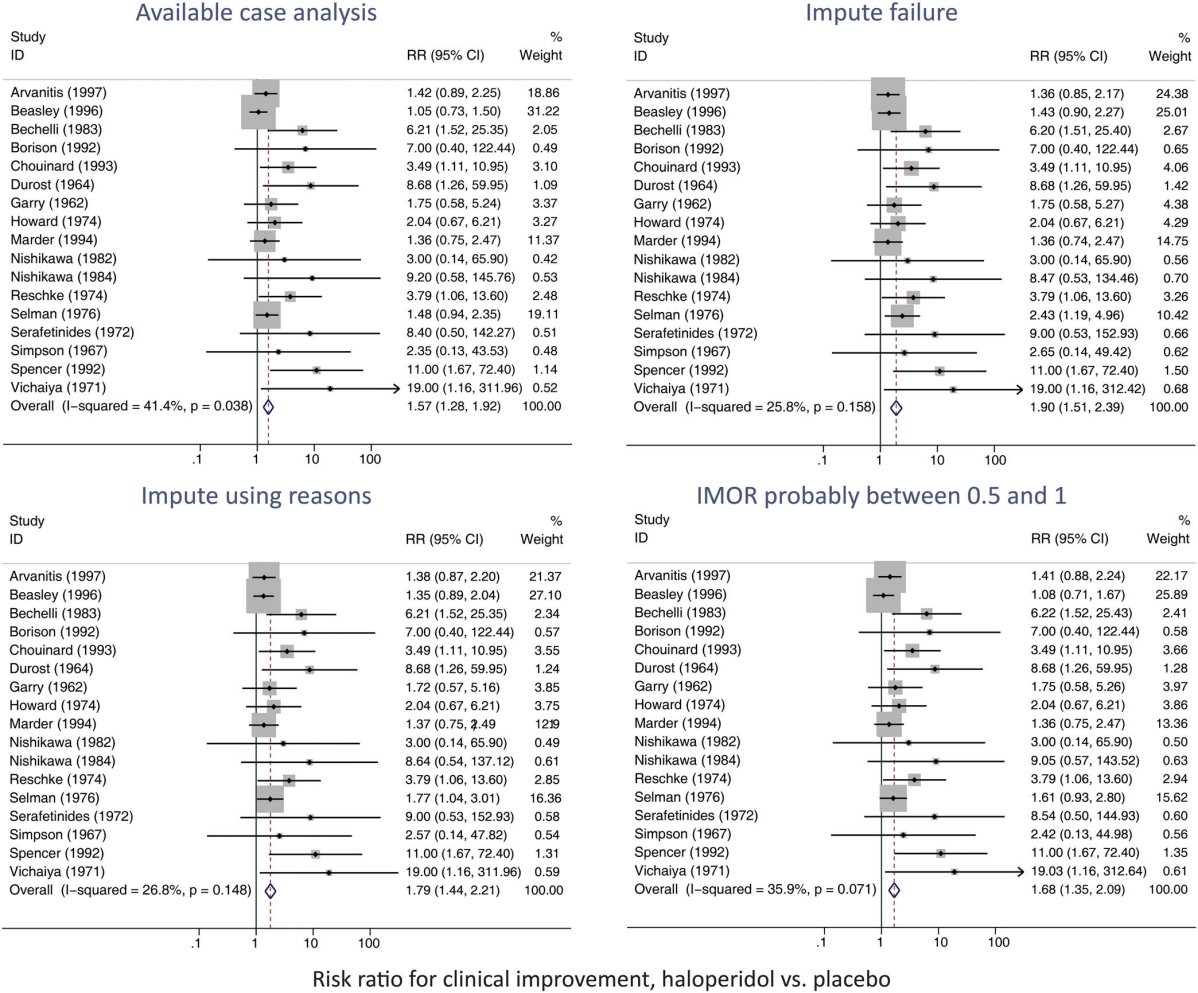
Haloperidol meta‐analysis under four different assumptions about the missing data [Colour figure can be viewed at http://wileyonlinelibrary.com]

Figure 2 shows the results of the four analyses. We first look at the study‐specific estimates listed under “RR (95% CI)” for the Beasley and Selman studies, which have substantial amounts of missing data and more missing data in the placebo arm (Table 2). Compared with the ACA analysis, the ICA‐0 analysis tends to impute more failures in the placebo arm and therefore gives larger estimated risk ratios for these studies. The confidence intervals widen because uncertainty for the risk ratio increases with lower risk, outweighing the benefit of increased sample size; for other measures such as the odds ratio, the confidence interval would narrow. The analysis using reasons imputes some but not all missing values as failures and therefore gives smaller increases in the estimated risk ratios and confidence interval widths. The analysis using IMORs imputes the missing values as slightly more likely to be failures than the ACA analysis and so slightly increases the estimated risk ratios, while the added uncertainty widens the confidence intervals. For the other 15 studies, all four analyses give similar estimates.

The changes in confidence interval width reduce the weight given to the Beasley study from 31% (in the ACA analysis) to 25%‐27% in the other analyses and similarly reduce the weight for the Selman study from 19% to 10%‐16%. The reduction in weight given to the Beasley study is important because this study has a lower risk ratio than other studies. The meta‐analysis results in Figure 2 therefore show that the pooled estimate increases from 1.57 in the ACA analysis to 1.68 to 1.90 in the other analyses, with corresponding increases in confidence interval width.

### 6.2 Mirtazapine meta‐analysis

Our second example comprises eight studies comparing the effectiveness of mirtazapine and placebo in patients with major depression.^37^ The continuous outcome is the change in depression symptoms measured on a standardized rating scale. For both mirtazapine and placebo arms, we have the mean change, standard deviation, and numbers of patients with observed and missing data (Table 3). We synthesize the mean differences using a random‐effects model.

**Table 3:** Mirtazapine meta‐analysis: mean change in depression scores, standard deviations (SDs), and numbers of observed and missing outcomes for the mirtazapine and placebo arms

Study	Mirtazapine Arm				Placebo Arm			
	Mean	SD	Observed	Missing	Mean	SD	Observed	Missing
Claghorn 1995	−14.5	8.8	26	19	−11.4	10.2	19	26
MIR 003‐003	−14.0	7.3	27	18	−11.5	8.3	24	21
MIR 003‐008	−12.6	8.0	23	37	−11.4	8.0	17	13
MIR 003‐020	−13.0	9.0	23	21	−6.2	6.5	24	19
MIR 003‐021	−13.8	5.9	22	28	−17.4	5.3	21	29
MIR 003‐024	−15.7	6.7	30	20	−11.1	9.9	27	23
MIR 84023a	−14.2	7.6	35	25	−11.9	8.6	33	24
MIR 84023b	−14.7	8.4	51	13	−11.8	8.3	48	18

**Figure 3:**
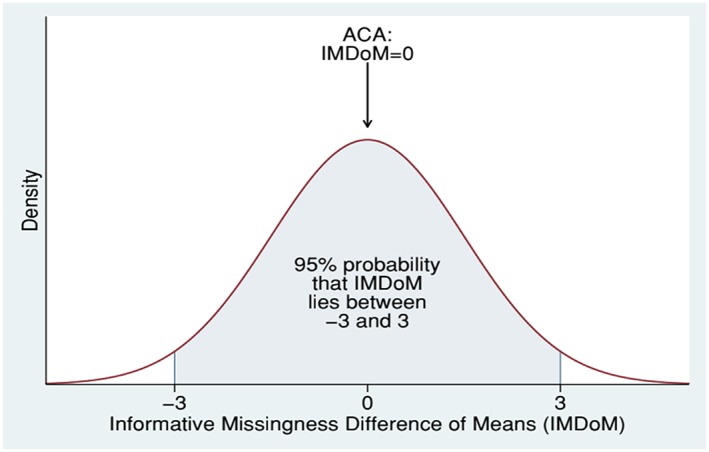
Plausible distribution for the informative missingness difference of means (IMDoM) in the mirtazapine meta‐analysis [Colour figure can be viewed at http://wileyonlinelibrary.com]

We present two of the possible ways to handle the missing data. ACA is the starting point in the analysis. As an alternative, we employ a pattern‐mixture model and use a plausible distribution for the IMDoM, in which the IMDoM is considered to lie between −3 and 3 with 95% probability (Figure 3). This implies that the mean value of IMDoM is zero and its standard deviation equal to 1.5. We chose this distribution to reflect a conservative scenario in which we do not believe that data are strictly MAR, but we allow for small departures from MAR that are equally likely in both directions. It is a conservative approach and a sound sensitivity analysis to assume a normal distribution for IMDoM centered at zero with a standard deviation allowing for some changes in the mean outcome between missing and observed participants. The magnitude of the standard deviation should depend on the scale used and the differences we expect to see in that scale.

**Figure 4:**
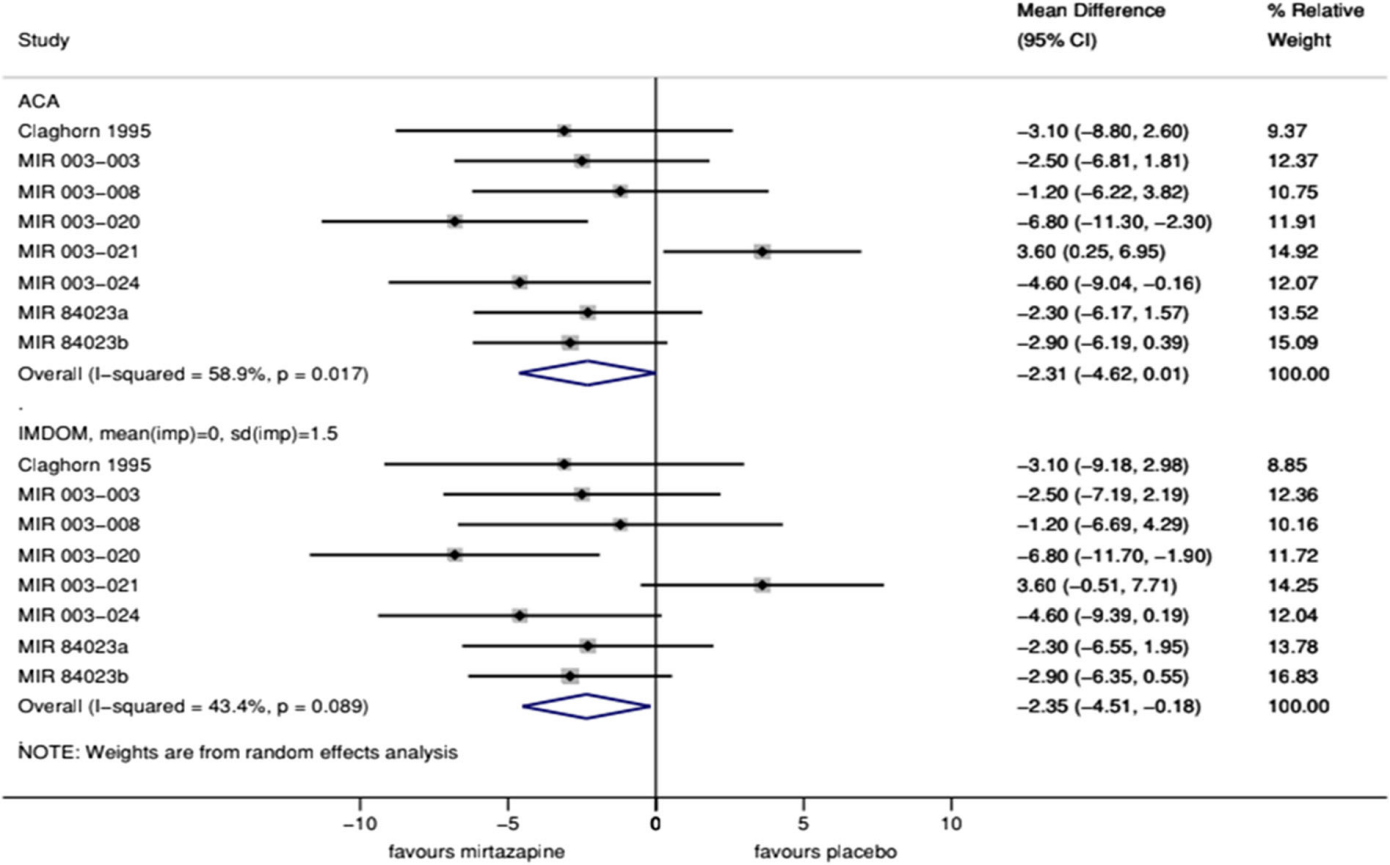
Mirtazapine meta‐analysis under two different assumptions about the missing data [Colour figure can be viewed at http://wileyonlinelibrary.com]

Figure 4 shows the results. Both methods give the same point estimate for the individual studies, because the IMDoM distribution in the MNAR analysis is centered at zero (its value in the MAR analysis). Study‐specific confidence intervals are wider for MNAR than MAR analyses, by 5% to 10% in most studies, but by 23% in the fifth study (MIR 003‐021), which has a larger proportion of missing data (Table 3). The MNAR analysis therefore assigns slightly smaller weight to MIR 003‐021; since this is the only study favoring placebo, the summary estimate shifts slightly towards mirtazapine and the heterogeneity variance declines (reflected in the decreased *I* ^2^ value and the narrower confidence interval about the summary estimate).

All these analyses may be performed using our software for Stata, available from the Statistical Software Components (SSC) archive. For binary outcomes, the IMOR approach and the approach using reasons are implemented in the metamiss command.^38^ For continuous outcomes, the IMDoM and IMRoM approaches are implemented in the metamiss2 command.^39^ A practical exercise using the haloperidol and mirtazapine data is described in Section 6. The solutions can be found at http://mtm.uoi.gr/index.php/meta-analysis-methods-and-tools.

## 7 DISCUSSION

In this manuscript, we reviewed and provided recommendations about missing outcome data for use in planning, conducting, meta‐analyzing, and reporting results from a systematic review.

Trial investigators should report the numbers of missing participants and results before imputation, even if they go on to impute missing data, and they should collect and report the reasons for dropout by trial arm.

In planning a systematic review, reviewers should consider the possibility of missing outcome data and plan to extract data about numbers of missing values and their reasons.

In conducting a systematic review, reviewers should be alert to the possibility that missing values have already been imputed and should aim to extract the unimputed data so that alternative imputation approaches can be used. The methodology presented in Section 5 has been extended to allow for imputed outcomes.^40^

In performing a meta‐analysis, a simple analysis such as ACA or ICA‐0 will often be used as a main analysis, but the more sophisticated methods described above form important sensitivity analyses.^28^ These should involve one or more analyses that make plausible assumptions about the missing data. The sensitivity analyses are typically specified after the systematic review, so that the nature of the trials can inform the plausible assumptions. With large amounts of missing data, results can be adjusted in so many ways that it would be difficult to know which estimates to believe. Hence, it is sensible to define the relevant sensitivity analyses a priori, in order to avoid the risk of data dredging. For example, if rich data on reasons are available, then imputation strategies should be defined for each reported reason; alternatively, background knowledge should be used to specify a plausible range of IMORs and hence to define an uncertainty approach. More suggestions for the uncertainty approach were given by White et al.^30^ We can apply the IMP models in a fully Bayesian framework using Monte Carlo to sample from the posterior distributions (eg, outcome in each arm).^31^, ^34^, ^35^ This is computationally slow, and we can alternatively use prior beliefs about the IMPs to inflate the observed standard errors of effect estimates and then proceed to their synthesis via meta‐analysis.^28^, ^30^, ^31^

Further research is needed in developing questionnaires to elicit values of the IMOR, IMDoM, or IMRoM^41^; in developing statistical methods allowing reason‐specific IMPs with uncertainty; and in developing methods for using reasons for missing data with continuous outcomes.

## FUNDING

Dimitris Mavridis is funded by the European Union's Horizon 2020 Framework Programme (no. 754936). I.W. was supported by the Medical Research Council Unit Programme number MC_UU_12023/21.

## CONFLICT OF INTEREST

The author reported no conflict of interest.

## DATA AVAILABILITY STATEMENT

All data are presented in the Tables (Tables 2 and 3), and anyone can use them.
